# The Functional Genetics of Handedness and Language Lateralization: Insights from Gene Ontology, Pathway and Disease Association Analyses

**DOI:** 10.3389/fpsyg.2017.01144

**Published:** 2017-07-06

**Authors:** Judith Schmitz, Stephanie Lor, Rena Klose, Onur Güntürkün, Sebastian Ocklenburg

**Affiliations:** Department of Biopsychology, Institute of Cognitive Neuroscience, Faculty of Psychology, Ruhr-University BochumBochum, Germany

**Keywords:** handedness, language lateralization, ontogenesis, gene ontology, asymmetry, genetics

## Abstract

Handedness and language lateralization are partially determined by genetic influences. It has been estimated that at least 40 (and potentially more) possibly interacting genes may influence the ontogenesis of hemispheric asymmetries. Recently, it has been suggested that analyzing the genetics of hemispheric asymmetries on the level of gene ontology sets, rather than at the level of individual genes, might be more informative for understanding the underlying functional cascades. Here, we performed gene ontology, pathway and disease association analyses on genes that have previously been associated with handedness and language lateralization. Significant gene ontology sets for handedness were anatomical structure development, pattern specification (especially asymmetry formation) and biological regulation. Pathway analysis highlighted the importance of the TGF-beta signaling pathway for handedness ontogenesis. Significant gene ontology sets for language lateralization were responses to different stimuli, nervous system development, transport, signaling, and biological regulation. Despite the fact that some authors assume that handedness and language lateralization share a common ontogenetic basis, gene ontology sets barely overlap between phenotypes. Compared to genes involved in handedness, which mostly contribute to structural development, genes involved in language lateralization rather contribute to activity-dependent cognitive processes. Disease association analysis revealed associations of genes involved in handedness with diseases affecting the whole body, while genes involved in language lateralization were specifically engaged in mental and neurological diseases. These findings further support the idea that handedness and language lateralization are ontogenetically independent, complex phenotypes.

## Introduction

Handedness and language lateralization are complex phenotypes and represent different aspects of functional brain asymmetries. Hemispheric asymmetries are a major principle of brain organization in many vertebrate (Ocklenburg et al., 2013d; Ströckens et al., 2013; Güntürkün and Ocklenburg, 2017) and invertebrate species (Frasnelli, 2013). In humans, handedness and language lateralization are related to some extent. Both are mostly controlled for by the left hemisphere in right-handed individuals. Moreover, left-handedness is associated with a higher probability for right-hemispheric language lateralization (Knecht et al., 2000; Somers et al., 2015). The predominance of the left hemisphere in processing fast temporal changes makes it ideally suited to process both complex motor function (Barber et al., 2012) and language (Slevc et al., 2011; Scott and McGettigan, 2013). This association prompted some authors to assume that one single gene determines both handedness and language lateralization: For example, the ‘Right-Shift Theory’ (Annett, 1975) proposes a single dominant allele (RS+), which increases the chance of being right-handed with a left-hemispheric dominance for language. The alternative recessive allele (RS-) does not influence lateralization, which reduces the ‘right-shift’ in RS+- individuals. In homozygous RS-- individuals, the direction of handedness and language lateralization is determined by chance. A similar single gene model has been conceived by McManus (1984, 1985), who proposed a dextral allele (D), which results in 100% right-handedness and left-hemispheric language dominance in homozygotes (DD). The chance allele (C) does not affect lateralization, so that right- and left-handedness occur with a probability of 50% each in the homozygote variant (CC). The heterozygote phenotype (DC) was proposed to result in a 75% probability of right-handedness. However, these early genetic theories are solely phenotype-driven and are not supported by molecular genetic evidence. In contrast, a number of twin studies estimated that around 25% of variance in handedness data is due to additive genetic effects. The remainder is suggested to be influenced by non-genetic factors (Medland et al., 2006, 2009; Vuoksimaa et al., 2009). In fact, no single gene has been identified as a potential exclusive determinant of handedness and language lateralization. Despite sample sizes allowing for adequate statistical power, evidence from genome-wide association studies (GWASs) strongly argues against the existence of such a gene (Eriksson et al., 2010; Ocklenburg et al., 2013c; Armour et al., 2014). However, these studies do not disprove the existence of a genetic component in handedness development *per se*. As suggested by McManus et al. (2013), a key biological model for the genetics of handedness is primary ciliary dyskinesia (PCD), which results in situs inversus, a mirror reversal of visceral organs, in 50% of all cases. Not surprisingly for a complex phenotype, at least 16 loci involved in PCD have been found so far. Similarly, molecular genetic studies suggest that multi-locus models might be a more suitable explanation for the ontogenesis of hemispheric asymmetries. Armour et al. (2014) suggest that at least 40 and potentially up to 100 genes are involved in the determination of functional lateralization.

Genes associated with handedness include *LRRTM1* (Francks et al., 2007), *PCSK6* (Scerri et al., 2011; Arning et al., 2013; Brandler et al., 2013; Robinson et al., 2016), *AR* (Medland et al., 2005; Hampson and Sankar, 2012; Arning et al., 2015), *COMT* (Savitz et al., 2007), *APOE* (Bloss et al., 2010; but see Hubacek et al., 2013; Piper et al., 2013), and *SETDB2* (Ocklenburg et al., 2015a). Genes associated with language lateralization include *FOXP2* (Pinel et al., 2012; Ocklenburg et al., 2013b), *CCKAR* (Ocklenburg et al., 2013a), *GRIN2B* (Ocklenburg et al., 2011), and others (see below). However, these genes explain only a fraction of the variance in the respective phenotype. To this date, no study could reveal an association of one gene with both language lateralization and handedness that would point towards a shared genetic basis. Therefore, Ocklenburg et al. (2014) proposed that handedness and language lateralization differ in both their neurophysiological basis and genetic correlates. The authors suggest a relationship of partial pleiotropy between both phenotypes, i.e., handedness and language lateralization have shared as well as independent ontogenetic influencing factors contributing to their development.

Uncovering the ontogenesis of hemispheric asymmetries requires deeper knowledge of genes involved in their development. However, specifically investigating individual genes gives rise to different methodological difficulties: First, genes can never be interpreted on their own, but have to be regarded in the context of other genes (Zhang et al., 2015) and environmental factors (Asor and Ben-Shachar, 2016; Gattere et al., 2016). Second, another promising way to shed light on the development of hemispheric asymmetries is comparing gene expression between the left and right hemisphere. Grouping of genes into functional sets could manifest hemispheric asymmetries that are too subtle to uncover on the level of individual genes (Karlebach and Francks, 2015). Accordingly, gene ontology (GO) sets classify genes into functional groups depending on their biological effects. Applying GO analysis on a certain list of genes reveals information on shared molecular functions of these genes, their contributions to biological processes and their corresponding cellular locations (Gene Ontology Consortium, 2015). Here, we applied GO analyses on genes previously associated with handedness on the one hand and genes previously associated with language lateralization on the other hand to identify functional gene groups associated with the respective phenotype. We hypothesized that functional gene groups between phenotypes are mainly independent from each other. This study will provide additional evidence opposing models that assume 100% pleiotropy (the same ontogenetic factors determine both handedness and language lateralization), but instead is in line with a model of partial pleiotropy (shared and individual ontogenetic factors determine handedness and language lateralization) as suggested by Ocklenburg et al. (2014).

## Materials and Methods

### Identification of Relevant Genes

In order to identify genes associated with handedness or language lateralization, we performed literature search using the database PubMed^1^. Molecular genetic studies were included if performed on human subjects.

We included individual genes previously identified in candidate gene studies on handedness or language lateralization into analysis (Medland et al., 2005; Francks et al., 2007; Bloss et al., 2010; Ocklenburg et al., 2011, 2013a,b; Hampson and Sankar, 2012; Pinel et al., 2012; Arning et al., 2013, 2015; Robinson et al., 2016). Furthermore, we included all genes reaching *p* < 10^-5^ in a GWAS by Scerri et al. (2011) and a GWAS meta-analysis by Brandler et al. (2013). We further included differentially expressed genes from gene expression studies (*p* < 0.01; Sun et al., 2005; Karlebach and Francks, 2015) and top hits identified by family-based genetic association analysis (Savitz et al., 2007) and manual segregation analysis (van Agtmael et al., 2002). Lastly, we included all genes with LOD > 1.5 from a linkage analysis published by Somers et al. (2015). **Table 1** shows the list of 63 genes previously associated with handedness ontogenesis. The list of 45 genes previously associated with the formation of language lateralization is listed in **Table 2**. Importantly, most of these genes do not reach conventional levels of significance or do not replicate. However, it is still likely that GO analysis reveals certain clusters of genes contributing to each of the phenotypes.

**Table 1:** Identified genes involved in handedness ontogenesis.

Gene	Type of association	Reference
*Activin receptor type-2B (ACVR2B)*	Genome-wide study meta-analysis	Brandler et al., 2013
*ADAMTS like 1 (ADAMTSL1)*	Genome-wide study meta-analysis	Brandler et al., 2013
*Androgen receptor gene* (*AR)*	Candidate gene study	Arning et al., 2015
	Candidate gene study	Hampson and Sankar, 2012
	Candidate gene study	Medland et al., 2005
*Androglobin (ADGB)*	Genome-wide study meta-analysis	Brandler et al., 2013
*Apolipoprotein E (APOE)*	Candidate gene study	Bloss et al., 2010
*ATP/GTP binding protein like 1 (AGBL1)*	Genome-wide association study	Scerri et al., 2011
*Breast carcinoma amplified sequence 1 (BCAS1)*	Genome-wide association study	Scerri et al., 2011
*Calcium voltage-gated channel auxiliary subunit alpha2delta 1 (CACNA2D1)*	Genome-wide study meta-analysis	Brandler et al., 2013
*Catechol-O-methyltransferase (COMT)*	Family-based genetic association analysis	Savitz et al., 2007
*Centromere protein C (CENPC1)*	Genome-wide study meta-analysis	Brandler et al., 2013
*Ceramide kinase (CERK)*	Genome-wide study meta-analysis	Brandler et al., 2013
*Chromosome 3 open reading frame 20 (C3orf20)*	Genome-wide association study	Scerri et al., 2011
*Coiled-coil domain containing 102B (CCDC102B)*	Genome-wide association study	Scerri et al., 2011
*C-type lectin domain family 3 member B (CLEC3B)*	Genome-wide association study	Scerri et al., 2011
*Dynein, axonemal, heavy chain 13 (DNAHC13)*	Manual allele sharing analysis	van Agtmael et al., 2002
*E2F transcription factor 8 (E2F8)*	Genome-wide study meta-analysis	Brandler et al., 2013
*Exosome component 7 (EXOSC7)*	Genome-wide association study	Scerri et al., 2011
*Feline leukemia virus subgroup C cellular receptor 1 (FLVCR1)*	Genome-wide association study	Scerri et al., 2011
*Frizzled class receptor 1 (FZD1)*	Genome-wide study meta-analysis	Brandler et al., 2013
*Fructose-bisphosphatase 2 (FBP2)*	Genome-wide association study	Scerri et al., 2011
*G protein-coupled receptor kinase 5 (GRK5)*	Genome-wide study meta-analysis	Brandler et al., 2013
*Gap junction protein alpha 1 (GJA1)*	Genome-wide study meta-analysis	Brandler et al., 2013
*GLI family zinc finger 3 (GLI3)*	Genome-wide study meta-analysis	Brandler et al., 2013
*Glypican 3 (GPC3)*	Genome-wide study meta-analysis	Brandler et al., 2013
*GTP binding protein 10 (GTPBP10)*	Genome-wide study meta-analysis	Brandler et al., 2013
*Integrin subunit beta 8 (ITGB8)*	Genome-wide association study	Scerri et al., 2011
*Laminin subunit alpha 5 (LAMA5)*	Genome-wide study meta-analysis	Brandler et al., 2013
*Latent transforming growth factor beta binding protein 1 (LTBP1)*	Genome-wide study meta-analysis	Brandler et al., 2013
*Leucine rich repeat transmembrane neuronal 1 (LRRTM1)*	Candidate gene study	Francks et al., 2007
*LIM domain only 4 (LMO4)*	Gene expression study (fetal cortex)	Sun et al., 2005
*LOC100132083*	Genome-wide association study	Scerri et al., 2011
*LOC441204*	Genome-wide study meta-analysis	Brandler et al., 2013
*Mahogunin ring finger 1 (MGRN1)*	Genome-wide study meta-analysis	Brandler et al., 2013
*Meiosis specific nuclear structural 1 (MNS1)*	Genome-wide study meta-analysis	Brandler et al., 2013
*Membrane associated guanylate kinase, WW and PDZ domain containing 1 (MAGI1)*	Genome-wide study meta-analysis	Brandler et al., 2013
*Microtubule associated scaffold protein 1 (MTUS1)*	Genome-wide association study	Scerri et al., 2011
*Neogenin 1 (NEO1)*	Genome-wide association study	Scerri et al., 2011
*Neuromedin B receptor (NMBR)*	Genome-wide association study	Scerri et al., 2011
*Nodal growth differentiation factor (NODAL)*	Manual allele sharing analysis	van Agtmael et al., 2002
*Pleiotrophin (PTN)*	Genome-wide association study	Scerri et al., 2011
*Polycystic kidney disease 2 (PKD2)*	Genome-wide study meta-analysis	Brandler et al., 2013
*Potassium channel tetramerization domain containing 18 (KCTD18)*	Genome-wide association study	Scerri et al., 2011
*Potassium sodium-activated channel subfamily T member 2 (KCNT2)*	Genome-wide study meta-analysis	Brandler et al., 2013
*Prolyl endopeptidase (PREP)*	Genome-wide study meta-analysis	Brandler et al., 2013
*Proprotein convertase subtilisin/kexin type 6 (PCSK6)*	Candidate gene study	Arning et al., 2013
	Genome-wide study meta-analysis	Brandler et al., 2013
	Candidate gene study	Robinson et al., 2016
	Genome-wide association study	Scerri et al., 2011
*RAB11 family interacting protein 4 (RAB11FIP4)*	Genome-wide study meta-analysis	Brandler et al., 2013
*Ras responsive element binding protein 1 (RREB1/HNT)*	Genome-wide association study	Scerri et al., 2011
*Regulatory factor X3 (RFX3)*	Genome-wide study meta-analysis	Brandler et al., 2013
*Replication protein A1 (RPA1)*	Genome-wide study meta-analysis	Brandler et al., 2013
*Retinoic acid receptor alpha (RARA)*	Genome-wide study meta-analysis	Brandler et al., 2013
*Ribosomal RNA processing 15 homolog (RRP15)*	Genome-wide association study	Scerri et al., 2011
*SET domain bifurcated 2 (SETDB2)*	Candidate gene study	Ocklenburg et al., 2015a
*Signal transducing adaptor family member 1 (STAP1)*	Genome-wide study meta-analysis	Brandler et al., 2013
*Tachykinin receptor 1 (TACR1)*	Genome-wide study meta-analysis	Brandler et al., 2013
*Teneurin transmembrane protein 3 (TENM1/ODZ3)*	Genome-wide association study	Scerri et al., 2011
*Thrombospondin type 1 domain containing 4 (THSD4)*	Genome-wide study meta-analysis	Brandler et al., 2013
*Transketolase (TKT)*	Genome-wide study meta-analysis	Brandler et al., 2013
*Transmembrane protein 87B (TMEM87B)*	Genome-wide study meta-analysis	Brandler et al., 2013
*Tryptophan hydroxylase 2 (TPH2)*	Genome-wide association study	Scerri et al., 2011
*Tumor protein p63 (TP63)*	Genome-wide study meta-analysis	Brandler et al., 2013
*UDP glucuronosyltransferase family 2 member B4 (UGT2B4)*	Genome-wide study meta-analysis	Brandler et al., 2013
*Vesicle trafficking 1 (VTA1)*	Genome-wide association study	Scerri et al., 2011
*Zinc finger protein 385D (ZNF385D)*	Genome-wide study meta-analysis	Brandler et al., 2013

**Table 2:** Identified genes involved in the ontogenesis of language lateralization.

Gene	Type of association	Reference
*5-hydroxytryptamine receptor 1B (HTR1B)*	Gene expression study (adult cortex)	Karlebach and Francks, 2015
*ADAM metallopeptidase with thrombospondin type 1 motif 4 (ADAMTS4)*	Gene expression study (adult cortex)	Karlebach and Francks, 2015
*BMP/retinoic acid inducible neural specific 1 (BRINP1)*	Linkage analysis	Somers et al., 2015
*Cancer susceptibility candidate 15 (CASC15)*	Linkage analysis	Somers et al., 2015
*Carboxypeptidase A2 (CPA2)*	Linkage analysis	Somers et al., 2015
*CCR4-NOT transcription complex subunit 4 (CNOT4)*	Linkage analysis	Somers et al., 2015
*Chloride voltage-gated channel 1 (CLCN1)*	Linkage analysis	Somers et al., 2015
*Cholecystokinin A receptor (CCKAR)*	Candidate gene study	Ocklenburg et al., 2013a
*Chromosome 1 open reading frame 95 (C1orf95)*	Gene expression study (adult cortex)	Karlebach and Francks, 2015
*Chromosome 14 open reading frame 132 (C14orf132)*	Gene expression study (adult cortex)	Karlebach and Francks, 2015
*Chromosome 6 open reading frame 142 (C6orf142)*	Gene expression study (adult cortex)	Karlebach and Francks, 2015
*Cytochrome P450 family 27 subfamily A member 1 (CYP27A1)*	Gene expression study (adult cortex)	Karlebach and Francks, 2015
*Deleted in esophageal cancer 1 (DEC1)*	Linkage analysis	Somers et al., 2015
*Diaphanous related formin 2 (DIAPH2)*	Gene expression study (adult cortex)	Karlebach and Francks, 2015
*Dopamine receptor D2 (DRD2)*	Gene expression study (adult cortex)	Karlebach and Francks, 2015
*EPH receptor A6 (EPHA6)*	Gene expression study (adult cortex)	Karlebach and Francks, 2015
*Family with sequence similarity 65, member B (FAM65B)*	Gene expression study (adult cortex)	Karlebach and Francks, 2015
*Forkhead box P2 (FOXP2)*	Candidate gene study	Ocklenburg et al., 2013b
	Candidate gene study	Pinel et al., 2012
*Galanin and GMAP prepropeptide (GAL)*	Gene expression study (adult cortex)	Karlebach and Francks, 2015
*Glutamate ionotropic receptor kainate type subunit 2 (GRIK2)*	Gene expression study (adult cortex)	Karlebach and Francks, 2015
*Glutamate ionotropic receptor NMDA type subunit 2B (GRIN2B)*	Candidate gene study	Ocklenburg et al., 2011
*Glycine receptor alpha 2 (GLRA2)*	Gene expression study (adult cortex)	Karlebach and Francks, 2015
*Glypican 4 (GPC4)*	Gene expression study (adult cortex)	Karlebach and Francks, 2015
*Hippocalcin (HPCA)*	Gene expression study (adult cortex)	Karlebach and Francks, 2015
*Hyaluronan and proteoglycan link protein 4 (HAPLN4)*	Gene expression study (adult cortex)	Karlebach and Francks, 2015
*KIAA0319*	Candidate gene study	Pinel et al., 2012
*Long intergenic non-protein coding RNA, p53 induced transcript (LINC-PRINT)*	Linkage analysis	Somers et al., 2015
*Neurofilament heavy (NEFH)*	Gene expression study (adult cortex)	Karlebach and Francks, 2015
*Neuronal differentiation 1 (NEUROD1)*	Gene expression study (adult cortex)	Karlebach and Francks, 2015
*Nuclear receptor subfamily 2 group F member 2 (NR2F2)*	Gene expression study (adult cortex)	Karlebach and Francks, 2015
*Parvalbumin (PVALB)*	Gene expression study (adult cortex)	Karlebach and Francks, 2015
*Plexin C1 (PLXNC1)*	Gene expression study (adult cortex)	Karlebach and Francks, 2015
*Potassium channel tetramerization domain containing 4 (KCTD4)*	Gene expression study (adult cortex)	Karlebach and Francks, 2015
*Protein tyrosine phosphatase, non-receptor type 3 (PTPN3)*	Gene expression study (adult cortex)	Karlebach and Francks, 2015
*Protein tyrosine phosphatase, receptor type R (PTPRR)*	Gene expression study (adult cortex)	Karlebach and Francks, 2015
*Regulator of G-protein signaling 8 (RGS8)*	Gene expression study (adult cortex)	Karlebach and Francks, 2015
*RNA binding motif protein 33 (RBM33)*	Linkage analysis	Somers et al., 2015
*SGK2, serine/threonine kinase 2 (SGK2)*	Gene expression study (adult cortex)	Karlebach and Francks, 2015
*Sodium voltage-gated channel alpha subunit 3 (SCN3A)*	Gene expression study (adult cortex)	Karlebach and Francks, 2015
*Solute carrier family 6 member 9 (SLC6A9)*	Gene expression study (adult cortex)	Karlebach and Francks, 2015
*Synaptotagmin 2 (SYT2)*	Gene expression study (adult cortex)	Karlebach and Francks, 2015
*THEM2*	Candidate gene study	Pinel et al., 2012
*TTRAP*	Candidate gene study	Pinel et al., 2012
*Yippee like 1 (YPEL1)*	Gene expression study (adult cortex)	Karlebach and Francks, 2015
*Zinc finger CCHC-type containing 12 (ZCCHC12)*	Gene expression study (adult cortex)	Karlebach and Francks, 2015

### Gene Ontology Analysis

We used WebGestalt (WEB-based GEne SeT AnaLysis Toolkit) (Zhang et al., 2005; Wang et al., 2013) to identify shared functional groups of all genes associated with handedness (see **Table 1**). The list containing 63 genes was inserted to WebGestalt to identify GO sets associated with handedness. A GO set is a pre-defined list of genes that share either molecular functions (biochemical activity of a gene product), cellular components (place in the cell where a gene product is active), or biological processes (biological objective of a gene or gene product). For example, the GO set ‘determination of left/right symmetry’ contains 82 genes and gene products whose biological objective is involved in body formation in a symmetric or asymmetric pattern (Ashburner et al., 2000).

For each GO set, WebGestalt calculated a ratio of enrichment (RE) by comparing the observed number of genes in the inserted gene list and also in the GO set (O) to the expected number of genes in the inserted gene list and also in the GO set (E). This expected value (E) was based on the number of genes in the inserted gene list (L) multiplied with the number of genes in the GO set (GO) and divided by the number of genes in the reference gene set (RG). If the observed value (O) exceeded the expected value (E), the GO set was enriched with a ratio of enrichment RE = O/E (Wang et al., 2013). WebGestalt then used the hypergeometric test to evaluate the significance of enrichment for GO sets in the list of genes. The significance level was set to 0.05 after Benjamini–Hochberg correction for multiple comparisons (Benjamini and Hochberg, 1995). WebGestalt only reported GO sets with corrected *p*-values smaller than 0.05.

In addition to statistical results, WebGestalt’s output included a visualization of relationships between GO sets. This hierarchical structure of GO sets included high level GO sets representing broad molecular functions/cellular components/biological processes, e.g., ‘signal transduction (GO:0007165).’ These broader GO sets were subdivided into more specific lower level GO sets, e.g., ‘regulation of postsynaptic neurotransmitter receptor activity (GO:0098962)’ (Ashburner et al., 2000). In order to improve the results’ transparency, significant lower level GO sets were clustered in superordinate groups of high level GO sets by visual inspection of this hierarchical structure.

The same procedure was applied on the gene list containing 45 genes associated with ontogenesis of language lateralization (see **Table 2**).

### KEGG Pathway Analysis

Using WebGestalt, we performed KEGG (Kyoto Encyclopedia of Genes and Genomes) pathway analyses (Kanehisa et al., 2008) to identify biological pathways including genes associated with the gene list of either handedness or language lateralization. Each list of genes (see **Tables 1**, **2**) was entered to WebGestalt separately. KEGG pathways are pre-defined lists of genes that are involved in biological pathways. A RE was calculated for each KEGG pathway analogous to GO analysis. The significance of enrichment for each KEGG pathway was calculated with the hypergeometric test. The significance level was set to 0.05 after Benjamini–Hochberg correction for multiple comparisons (Benjamini and Hochberg, 1995).

### Disease Association Analysis

In order to identify diseases associated with gene sets involved in either handedness or language lateralization, we conducted disease association analyses using WebGestalt (Wang et al., 2013). Gene-disease associations were inferred using GLAD4U (Gene List Automatically Derived For You) (Jourquin et al., 2012). Both gene lists (see **Tables 1**, **2**) were entered to WebGestalt separately. A RE was calculated for each disease. The significance of enrichment was calculated using hypergeometric test with a significance level of 0.05 after Benjamini–Hochberg correction (Benjamini and Hochberg, 1995). Using ICD-10 (World Health Organization, 1992), we identified diseases categorized under “V: Mental and behavioral disorders” or “VI: Diseases of the nervous system” as disorders related to the central nervous system (CNS).

## Results

### Lower Level GO Sets Involved in Handedness and Language Lateralization

After correction for multiple comparisons, GO analysis revealed 64 significant lower level GO sets for the 63 genes associated with handedness, among them 40 biological processes (see **Table 3**), 20 molecular functions, and 4 cellular components (see **Supplementary Figure S1** for full hierarchical GO set overview). Top hits were ‘epithelial tube morphogenesis (GO:0060562)’ (*p* < 0.001), ‘tube development (GO:0035295)’ (*p* < 0.001), ‘tube morphogenesis (GO: 0035239)’ (*p* < 0.001) as well as ‘determination of left/right symmetry (GO:0007368)’/‘determination of bilateral symmetry (GO:0009855)’/‘specification of symmetry (GO:0009799)’ (all *p* < 0.001). GO sets with the most genes involved were ‘protein binding (GO:0005515)’ (*p* < 0.05) with 20 handedness genes involved and ‘anatomical structure development (GO:0048856)’ (*p* < 0.01) and ‘multicellular organismal development (GO:0007275)’ (*p* < 0.01) with 18 handedness genes involved.

**Table 3:** Lower level and high level gene ontology (GO) sets enriched in genes associated with handedness ontogenesis.

Lower level GO set	GO ID	Number of genes involved	*P*-value	High level GO set
Epithelial tube morphogenesis	GO:0060562	8	9.6 × 10^-6^	Anatomical structure development
Tube development	GO:0035295	9	2.2 × 10^-5^	
Tube morphogenesis	GO:0035239	8	2.2 × 10^-5^	
Morphogenesis of an epithelium	GO:0002009	8	6.7 × 10^-5^	
Circulatory system development	GO:0072359	10	7.3 × 10^-5^	
Cardiovascular system development	GO:0072358	10	7.3 × 10^-5^	
Embryonic morphogenesis	GO:0048598	8	0.0002	
Anatomical structure formation involved in morphogenesis	GO:0048646	13	0.0002	
Tissue morphogenesis	GO:0048729	8	0.0002	
Neural tube development	GO:0021915	5	0.0003	
Tissue development	GO:0009888	12	0.0003	
Heart development	GO:0007507	7	0.0003	
Embryo development	GO:0009790	10	0.0003	
Morphogenesis of embryonic epithelium	GO:0016331	5	0.0003	
Epithelium development	GO:0060429	8	0.0005	
Organ development	GO:0048513	15	0.0007	
Chordate embryonic development	GO:0043009	7	0.0012	
Ureteric bud development	GO:0001657	4	0.0012	
Vasculature development	GO:0001944	7	0.0012	
Anatomical structure morphogenesis	GO:0009653	13	0.0013	
Embryo development ending in birth or egg hatching	GO:0009792	7	0.0013	
System development	GO:0048731	17	0.0014	
Anatomical structure development	GO:0048856	18	0.0017	
Organ morphogenesis	GO:0009887	8	0.0017	
Artery development	GO:0060840	3	0.0017	
Determination of left/right symmetry	GO:0007368	5	6.7 × 10^-5^	Pattern specification
Determination of bilateral symmetry	GO:0009855	5	7.3 × 10^-5^	
Specification of symmetry	GO:0009799	5	7.3 × 10^-5^	
Pattern specification process	GO:0007389	7	0.0005	
Cell fate commitment	GO:0045165	5	0.0014	
Multicellular organismal development	GO:0007275	18	0.0017	
Determination of heart left/right asymmetry	GO:0061371	3	0.0017	
Regulation of cell differentiation	GO:0045595	10	0.0005	Biological regulation
Cell fate specification	GO:0001708	4	0.0005	
Regulation of embryonic development	GO:0045995	4	0.0006	
Regulation of developmental process	GO:0050793	11	0.0012	
Positive regulation of nitrogen compound metabolic process	GO:0051173	10	0.0013	
Regulation of protein import into nucleus	GO:0042306	4	0.0017	
Regulation of protein localization to nucleus	GO:1900180	4	0.0017	
Regulation of intracellular protein transport	GO:0033157	4	0.0029	

*P-values are corrected for multiple comparisons using Benjamini–Hochberg correction*.

For the 45 genes associated with language lateralization, GO analysis revealed 97 significant lower level GO sets. Among these GO sets were 40 biological processes (see **Table 4**), 29 molecular functions, and 28 cellular components (see **Supplementary Figure S2** for full hierarchical GO set overview). Top hits of GO sets were ‘negative regulation of synaptic transmission, glutamatergic (GO:0051967)’ (*p* < 0.001), ‘feeding behavior (GO:0007631)’ (*p* < 0.001), and ‘signal release (GO:0023061)’ (*p* < 0.01). Most genes were involved in the cellular components ‘plasma membrane (GO:0005886)’ (*p* < 0.05), ‘cell periphery (GO:0071944)’ (*p* < 0.05) with 17 genes each and in the biological process ‘nervous system development (GO:0007399)’ (*p* < 0.01) with 13 genes involved.

**Table 4:** Lower level and high level GO sets enriched in genes associated with the ontogenesis of language lateralization.

Lower level GO set	GO ID	Number of genes involved	*P*-value	High level GO set
Feeding behavior	GO:0007631	5	0.0005	Response to stimulus
Response to cocaine	GO:0042220	3	0.0024	
Response to tropane	GO:0014073	3	0.0024	
Auditory behavior	GO:0031223	2	0.0030	
Behavior	GO:0007610	7	0.0052	
Mechanosensory behavior	GO:0007638	2	0.0052	
Response to ammonium ion	GO:0060359	3	0.0052	
Startle response	GO:0001964	2	0.0127	
Behavioral defense response	GO:0002209	2	0.0132	
Learning	GO:0007612	3	0.0132	
Forebrain development	GO:0030900	6	0.0030	Nervous system development
Nervous system development	GO:0007399	13	0.0039	
Telencephalon development	GO:0021537	4	0.012	
G-protein coupled receptor internalization	GO:0002031	2	0.0074	Transport
Regulation of amine transport	GO:0051952	3	0.0094	
Regulation of dopamine secretion	GO:0014059	2	0.012	
Dopamine secretion	GO:0014046	2	0.012	
Growth hormone secretion	GO:0030252	2	0.012	
Insulin secretion	GO:0030073	4	0.012	
Peptide hormone secretion	GO:0030072	4	0.013	
Peptide secretion	GO:0002790	4	0.013	
Negative regulation of synaptic transmission, glutamatergic	GO:0051967	3	0.0004	Signaling
Signal release	GO:0023061	7	0.0019	
Generation of a signal involved in cell-cell signaling	GO:0003001	7	0.0019	
Regulation of transmission of nerve impulse	GO:0051969	5	0.0039	
Synaptic transmission, glutamatergic	GO:0035249	3	0.0052	
Negative regulation of G-protein coupled receptor protein signaling pathway	GO:0045744	3	0.011	
Negative adaptation of signaling pathway	GO:0022401	2	0.012	
Desensitization of G-protein coupled receptor protein signaling pathway	GO:0002029	2	0.01	
Adaptation of signaling pathway	GO:0023058	2	0.013	
Negative regulation of protein kinase B signaling cascade	GO:0051898	2	0.013	
Regulation of long-term neuronal synaptic plasticity	GO:0048169	3	0.0020	Biological regulation
Regulation of synaptic transmission, glutamatergic	GO:0051966	3	0.0030	
Negative regulation of synaptic transmission	GO:0050805	3	0.0039	
Negative regulation of transmission of nerve impulse	GO:0051970	3	0.0039	
Negative regulation of neurological system process	GO:0031645	3	0.0052	
Regulation of neuronal synaptic plasticity	GO:0048168	3	0.0052	
Regulation of neurological system process	GO:0031644	5	0.0052	
Regulation of system process	GO:0044057	6	0.012	
Regulation of synaptic transmission	GO:0050804	4	0.013	

*P-values are corrected for multiple comparisons using Benjamini–Hochberg correction*.

Two lower level GO sets concerning cellular components overlap between the gene lists for handedness and language lateralization: ‘cell projection (GO:0042995)’ (*p* < 0.05) and ‘neuron projection (GO:0043005)’ (*p* < 0.05). There was no overlap in biological processes.

The distribution of raw *p*-values for all significantly enriched GO sets for handedness and language lateralization is displayed in **Supplementary Figure S3**.

### High Level GO Sets Involved in Handedness and Language Lateralization

Visual inspection of the hierarchical relationship between GO sets involved in handedness revealed that significant lower level GO sets regarding biological processes are clustered into three high level GO sets. First, 25 enriched lower level GO sets are involved in anatomical structure development. ‘Epithelial tube morphogenesis (GO:0060562)’ was the most significantly enriched GO set overall. Lower level GO sets contain not only ‘neural tube development (GO:0021915),’ but also ‘cardiovascular system development (GO:0072358),’ ‘artery development (GO:0060840),’ and ‘ureteric bud development (GO:0001657).’ Moreover, 6 lower level GO sets involve pattern specification, for example in terms of ‘specification of symmetry (GO:0009799),’ ‘determination of left/right symmetry (GO:0007368),’ and ‘determination of bilateral symmetry (GO:0009855).’ Lastly, 9 lower level GO sets involve biological regulation. These GO sets include ‘regulation of developmental process (GO:0050793)’ and ‘regulation of cell differentiation (GO:0045595).’ High level GO sets for genes associated with handedness are visualized in **Supplementary Figure S4**.

In contrast, significant lower level GO sets regarding biological processes in language lateralization are clustered into five high level GO sets. First, 10 enriched lower level GO sets can be described by the high level GO set ‘response to stimuli.’ These GO sets range from ‘feeding behavior (GO:0007631)’ to external stimuli like ‘behavioral defense response (GO:0002209)’ or ‘learning (GO:0007612)’ and organic substances like ‘response to cocaine (GO:0042220).’ Second, 3 lower level GO sets are involved in the high level GO set ‘nervous system development (GO:0007399),’ more specifically ‘forebrain development (GO:0030900),’ ‘telencephalon development (GO:0021537),’ and ‘nervous system development (GO:0007399).’ The third high level GO set with 8 lower level GO sets describes different forms of transport like ‘dopamine secretion (GO:0014046),’ ‘insulin secretion (GO:0030073)’ or ‘regulation of amine transport (GO:0051952).’ The fourth high level GO set includes 10 lower level GO sets involved in signaling, for example ‘regulation of transmission of nerve impulse (GO:0051969)’ or ‘synaptic transmission, glutamatergic (GO:0035249).’ Lastly, 9 lower level GO sets describe biological regulation, for example ‘regulation of long-term neuronal synaptic plasticity (GO:0048169)’ and ‘regulation of neurological system process (GO:0031644).’ High level GO sets for genes involved in language lateralization are visualized in **Supplementary Figure S4**.

Among the high level GO sets, biological regulation is involved in both handedness and language lateralization (see **Supplementary Figure S4**).

### KEGG Pathway Analysis

For genes involved in handedness, KEGG analysis yielded six KEGG pathways significantly enriched after correction for multiple comparisons: ‘Pathways in cancer’ (*p* < 0.001), ‘Basal cell carcinoma’ (*p* < 0.01), ‘ECM-receptor interaction’ (*p* < 0.01), ‘TGF-beta signaling pathway’ (*p* < 0.01), ‘Cell adhesion molecules (CAMs)’ (*p* < 0.01), and ‘Focal adhesion’ (*p* < 0.05).

For genes involved in language lateralization, KEGG analysis yielded four KEGG pathways significantly enriched after correction for multiple comparisons: ‘Neuroactive ligand-receptor interaction’ (*p* < 0.001), ‘Amyotrophic lateral sclerosis (ALS)’ (*p* < 0.01), ‘Pancreatic secretion’ (*p* < 0.001), and ‘Axon guidance’ (*p* < 0.01). The distribution of corresponding raw *p*-values is displayed in **Supplementary Figure S3**.

### Disease Association Analysis

Genes associated to handedness ontogenesis were involved in 156 diseases, among them 61 CNS-related diseases (39.10%). The most significantly enriched diseases were ‘Craniofacial Abnormalities’ (*p* < 0.001), ‘Amnesia’ (*p* < 0.001), and ‘Bone Diseases, Developmental’ (*p* < 0.01). Most genes were involved in ‘Craniofacial Abnormalities’ (*p* < 0.001) and ‘Congenital Abnormalities’ (*p* < 0.01) (six genes involved) and ‘Gilbert Disease’ (*p* < 0.01), ‘Epithelial cancers’ (*p* < 0.01), ‘Musculoskeletal Abnormalities’ (*p* < 0.01), and ‘Cancer or viral infections’ (*p* < 0.05) with five genes involved.

Genes involved in language lateralization were mostly associated to CNS-related diseases. 81 of 94 (86.17%) significantly enriched diseases were involved in mental or psychiatric states. The disease categories ‘Mental Disorders’ (*p* < 0.001), ‘Substance-Related Disorders’ (*p* < 0.001), and ‘Alcoholism’ (*p* < 0.001) were most significantly enriched. ‘Mental Disorders’ (*p* < 0.001) was enriched with 10 genes involved in language lateralization, followed by ‘Substance-Related Disorders’ (*p* < 0.001) and ‘Nervous System Diseases’ (*p* < 0.001) with seven genes involved. Associations between diseases and gene lists were much stronger in terms of *p*-values for genes involved in language lateralization than for genes involved in handedness (see **Supplementary Figure S3**).

There was considerable overlap in the enriched diseases for genes involved in handedness and language lateralization. Forty-two diseases were involved in both phenotypes, among them 39 (92.86%) CNS-related diseases.

## Discussion

Handedness and language lateralization have been proposed to share a common ontogenetic basis (Annett, 1975), but single genes involved in the formation of both phenotypes have not been identified (Ocklenburg et al., 2014). Here we show that the GO sets enriched in language lateralization barely overlap with those found for handedness. Thus, in addition to the fact that individual genes involved in handedness and language lateralization development are independent from each other, functional gene products also differ fundamentally with no shared biological processes. This indicates different functional cascades underlying handedness and language lateralization.

For genes involved in ontogenesis of handedness, significant lower level GO sets of biological processes are clustered into three high level GO sets (see **Supplementary Figure S4**). First, most lower level GO sets describe anatomical structure development in different body parts. This implies that genes involved in handedness development exert their effect at an early embryonic stage and their functional gene products do not only contribute to the CNS, but also to the whole body. This is in line with the suggestion by Brandler et al. (2013), who claim that handedness is partially controlled by the molecular mechanisms that establish body asymmetry during early development. This finding has been supported by neuroimaging studies of patients with situs inversus, who displayed atypical patterns of frontal and occipital cerebral asymmetries (Kennedy et al., 1999; Ihara et al., 2010). However, situs inversus patients display the standard pattern of handedness, which rather supports a dissociation between visceral and brain asymmetries (Matsumoto et al., 1997; McManus et al., 2004; Afzelius and Stenram, 2006). It might be that genes associated with handedness are not necessarily involved in body asymmetry formation, but rather in anatomical structure development *per se*. Interestingly, most of the significant lower level GO sets involved in anatomical structure development include the *androgen receptor (AR)* gene. Prenatal testosterone has been shown to affect handedness and language lateralization in opposite directions (Lust et al., 2011). Our findings suggest that the capacity of binding testosterone in the developing fetal brain might induce differences in anatomical structure development that affect handedness, but not language lateralization. This finding is highly interesting in the context of sex differences in hemispheric asymmetries. While it is more or less undisputed that there is a 1.23 higher rate of male compared to female left-handers (Papadatou-Pastou et al., 2008), there are not necessarily sex differences in language lateralization (McManus, 2010). If that is the case, the findings from GO analysis may contribute to the explanation of this effect. Another high level GO set involved in handedness development is ‘pattern specification process (GO:0007389).’ As expected, the significant GO sets indicate the involvement of handedness genes on symmetry and asymmetry development. This result comes to no surprise, as there may likely be an ascertainment bias, since several of the original studies were candidate gene studies. Interestingly, KEGG pathway analysis revealed that genes involved in handedness ontogenesis are associated to the TGF-beta signaling pathway involved in bodily left-right asymmetry (Mittwoch, 2008; Shiratori and Hamada, 2014). While *ACVR2B* is involved in gonadal growth, embryo differentiation, and placenta formation, *NODAL* is involved in left-right axis determination and mesoderm and endoderm induction (see **Supplementary Figure S5**). This finding indicates an involvement of the TGF-beta signaling pathway on handedness ontogenesis at an early stage of development. In a recent study, asymmetrical gene expression was found between left and right human spinal cord at 8 weeks post conception. Besides DNA methylation patterns, gene expression asymmetries were epigenetically regulated by miRNAs involved in the TGF-beta signaling pathway. Since preliminary forms of handedness are already visible at this time point before the spinal cord and the motor cortex are functionally connected, the TGF-beta signaling pathway might have an impact on early behavioral asymmetries in arm movements (Ocklenburg et al., 2017). This in line with our finding that the TGF-beta signaling pathway is involved in handedness, but not in language lateralization. The last high level GO set of biological processes enriched in handedness genes is comprised of biological regulation, for example on developmental processes as well as cell differentiation. This indicates a regulatory function of genes associated with handedness on all levels of developmental control and cell fate determination.

For genes involved in ontogenesis of language lateralization, four high level GO sets were identified. Many lower level GO sets describe responses to different stimuli. Especially the role of the GO sets ‘startle response (GO:0001964)’ and ‘behavioral defense response (GO:0002209)’ are in line with a relation between stress and the ontogenesis of hemispheric asymmetries that has been reported in many vertebrate species (see Ocklenburg et al., 2016). It has been shown that both acute and chronic stress can affect different forms of lateralization in the human brain. Our findings here suggest that genetic predispositions for certain response patterns may also play a role in the ontogenesis of language lateralization, implying a role for gene-environment interactions during asymmetry development. Another highly interesting GO set involved in the formation of language lateralization is ‘learning (GO:0007612).’ Compared to handedness, language is more closely related to cognition, which is in line with the role of genes associated with language lateralization on neuronal signaling, e.g., neurotransmitters like glutamate and dopamine (Ocklenburg et al., 2011, 2013a). Also, the involvement of learning processes in the ontogenesis of language lateralization (Thomas et al., 1997) indicates a greater role of neuronal plasticity processes for this phenotype than for handedness. Secondly, lower level GO sets are involved in nervous system development. Compared to GO sets enriched in genes involved in handedness, which comprise cerebral, but also body development, this result suggests that genes involved in language lateralization are specifically engaged within the CNS. This is also supported by our finding that genes involved in language lateralization are significantly enriched in the axon guidance pathway including *EPHA6* and *PLXNC1*, two receptors involved in axonal outgrowth, repulsion and attraction (see **Supplementary Figure S6**). In addition to their effect on basic cell metabolic processes, genes associated with language lateralization seem to be involved in neuronal signaling. ‘Negative regulation of G-protein coupled receptor protein signaling pathway (GO:0045744)’ or ‘desensitization of G-protein coupled receptor protein signaling pathway (GO:0002029)’ are important lower level GO sets within this category. The G-protein coupled receptor protein signaling pathway has been identified as asymmetrically expressed in adult human language related areas: Superior Temporal Gyrus (STS) and Heschl’s Gyrus (HG). Moreover, in our study many GO sets are involved in transmission of nerve impulse, a GO set asymmetrically expressed in STS, but not in HG (Karlebach and Francks, 2015). Lastly, lower level GO sets significantly enriched in genes associated with language lateralization are involved in the high level GO set of biological regulation. Although individual GO sets of language lateralization and handedness do not overlap in terms of biological processes, biological regulation represents a high level GO set within genes involved in both phenotypes. This can be considered as a minimal overlap between biological processes of gene products involved in handedness and those involved in language lateralization.

Overall, gene lists for handedness and language lateralization resulted in similar numbers of enriched GO sets. However, the distribution of genes differed between phenotypes. For genes associated with handedness, there were many GO sets with 10 or more genes enriched in. Thus, products of genes involved in handedness formation seems to be less complex compared to products of genes involved in language lateralization. The latter are more heterogenous with maximally seven genes enriched in the same GO set (with the exception of ‘nervous system development (GO:0007399)’ with 13 genes enriched) and less strong associations in terms of *p*-values.

In contrast, associations between diseases and gene lists were much stronger for genes involved in language lateralization than for genes involved in handedness. For language lateralization, many disease categories were enriched with high numbers of genes involved, mostly categorized in mental and neurological diseases. Among the diseases significantly associated with genes involved in language lateralization are schizophrenia (Ocklenburg et al., 2013e, 2015b) and autism spectrum disorders (Knaus et al., 2010; Tager-Flusberg, 2016). Language lateralization seems more strongly connected to disorders of neurological system development, which is completely in line with our finding that associated genes are enriched in nervous system development rather than anatomical structure development. In contrast, genes associated with handedness ontogenesis are involved in diseases affecting the whole body, which supports our findings from GO analyses and the argumentation pointed out by Brandler et al. (2013). Among the significantly enriched diseases were many that had been associated with handedness before, specifically depression (Denny, 2009), bipolar disorder (Nowakowska et al., 2008), language and learning disorders (Geschwind and Behan, 1982), anxiety disorders (Logue et al., 2015), attention deficit hyperactivity disorder (Brandler and Paracchini, 2014), and schizophrenia (Hirnstein and Hugdahl, 2014).

Our results support the idea of a model of partial pleiotropy for handedness and language lateralization as suggested by Ocklenburg et al. (2014). However, biological and statistical issues remain to be solved: First, two or more lists of genes could result in different GO sets that might still be highly intercorrelated and therefore related to one another. However, this may rather concern low level GO sets. In our data, high level superordinate GO sets between phenotypes are distinct from each other, but this limitation should nonetheless be kept in mind. Second, since most of the included genes of both lists do not reach conventional levels of significance or do not replicate in association studies or GWASs we cannot rule out that statistical noise could have had an impact on the results. Low pleiotropy between genes associated with handedness and language lateralization could therefore partly represent measurement error.

Taken together, our findings further suggest that handedness and language lateralization are ontogenetically independent, complex phenotypes (Ocklenburg et al., 2014). Relative independence of these phenotypes has also recently been concluded in terms of genetic background (Corballis, 2017) as well as in terms of neuroanatomy (Króliczak et al., 2016). Compared to genes involved in handedness ontogenesis, which mostly contribute to structural development, genes involved in language lateralization rather contribute to activity-dependent cognitive processes partly associated to mental and neurological disorders. When searching for overlapping genetic contributions to the ontogenesis of these two traits, our results indicate that particularly genes within the high level GO set of ‘biological regulation’ may represent promising candidate genes. Revealing further candidate genes for handedness and language lateralization will not only contribute to important insights into the development of hemispheric asymmetries, but also to a better understanding of disorders related to atypical lateralization, e.g., schizophrenia (Levchenko et al., 2014).

## Author Contributions

JS performed data collection, analyzed data and wrote the manuscript, SL analyzed data, RK analyzed data, OG designed the study, and SO designed the study. All authors discussed the results and edited the manuscript.

## Conflict of Interest Statement

The authors declare that the research was conducted in the absence of any commercial or financial relationships that could be construed as a potential conflict of interest.

## Footnotes

1 https://www.ncbi.nlm.nih.gov/pubmed

## Supplementary Material

FIGURE S1 Full hierarchical GO set overview for genes involved in handedness ontogenesis.

FIGURE S2 Full hierarchical GO set overview for genes involved in the ontogenesis of language lateralization.

FIGURE S3 Distribution of raw *p*-values for all significant lower level GO sets involved in handedness and language lateralization.

FIGURE S4 High level GO sets involved in handedness and language lateralization.

FIGURE S5 Output of KEGG analysis for the TGF-beta signaling pathway. Genes involved in handedness ontogenesis are highlighted in red.

FIGURE S6 Output of KEGG analysis for the axon guiding pathway. Genes involved in language lateralization are highlighted in red.
