# Supplementary figures and images for: The Functional Genetics of Handedness and Language Lateralization: Insights from Gene Ontology, Pathway and Disease Association Analyses
Source: Front Psychol. 2017 Jul 6;8:1144. doi: 10.3389/fpsyg.2017.01144 (PMC5498560; doi:10.3389/fpsyg.2017.01144)

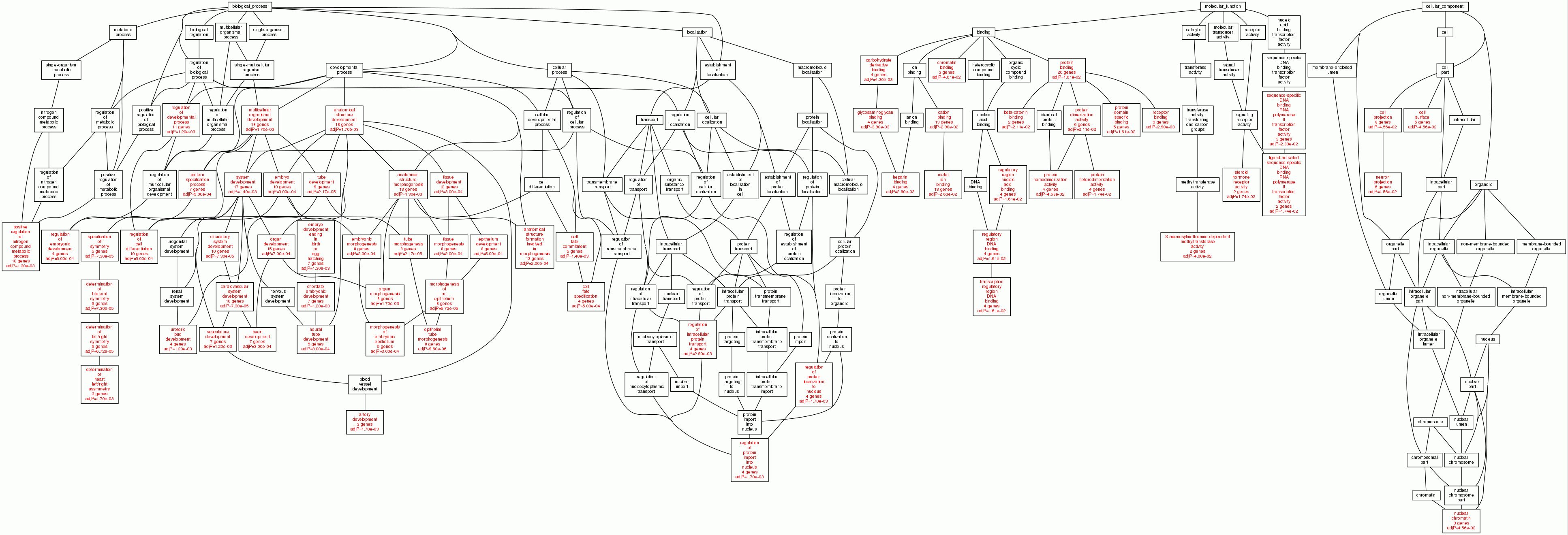

Supplement: FIGURE S1 — Full hierarchical GO set overview for genes involved in handedness ontogenesis. [file Image_1.JPEG]

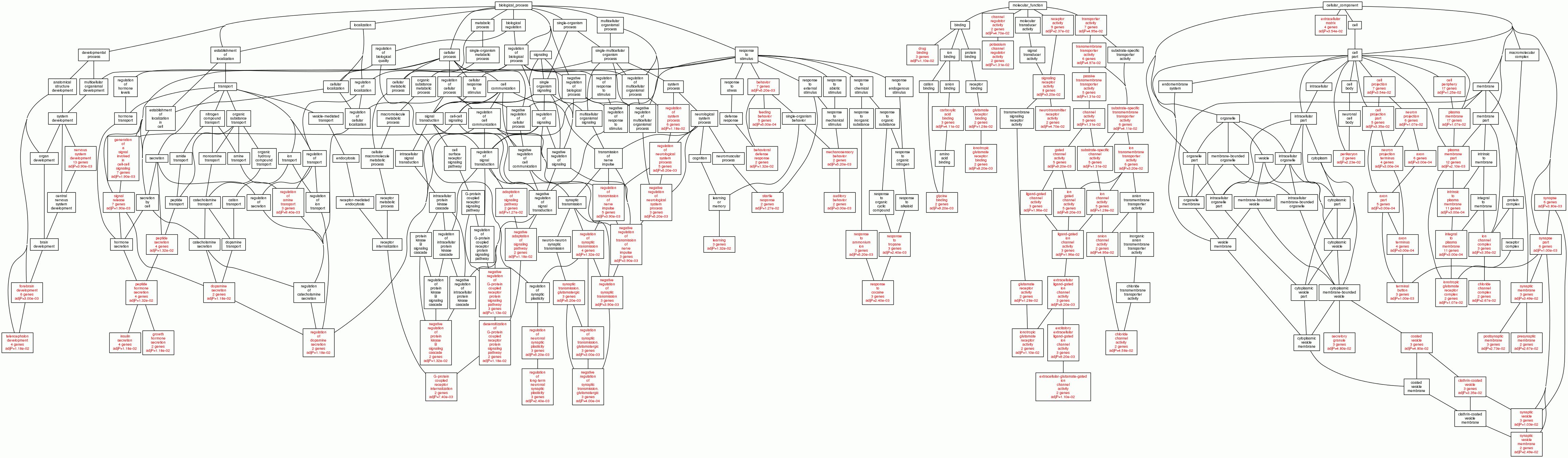

Supplement: FIGURE S2 — Full hierarchical GO set overview for genes involved in the ontogenesis of language lateralization. [file Image_2.JPEG]

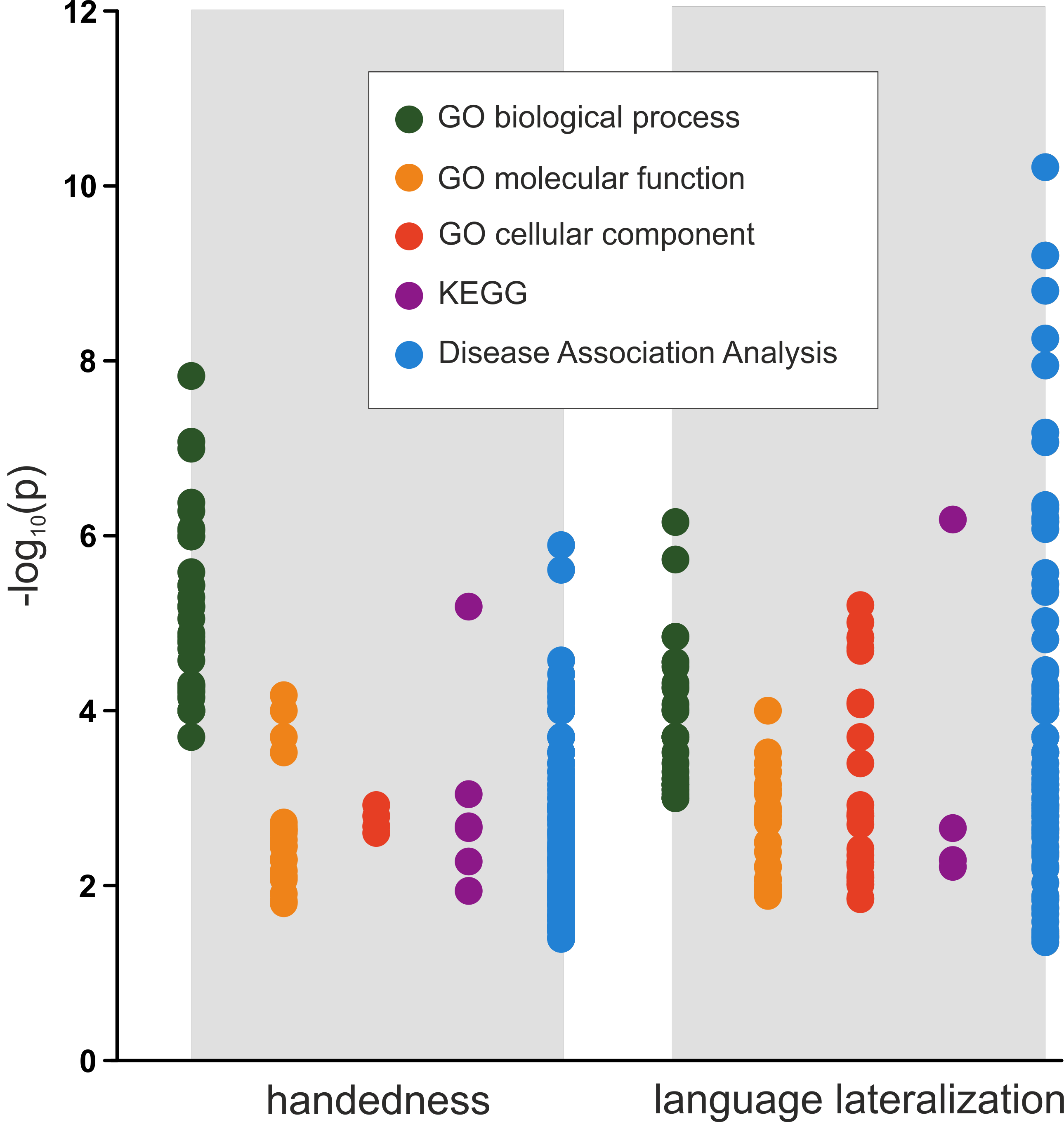

Supplement: FIGURE S3 — Distribution of raw p-values for all significant lower level GO sets involved in handedness and language lateralization. [file Image_3.TIF]

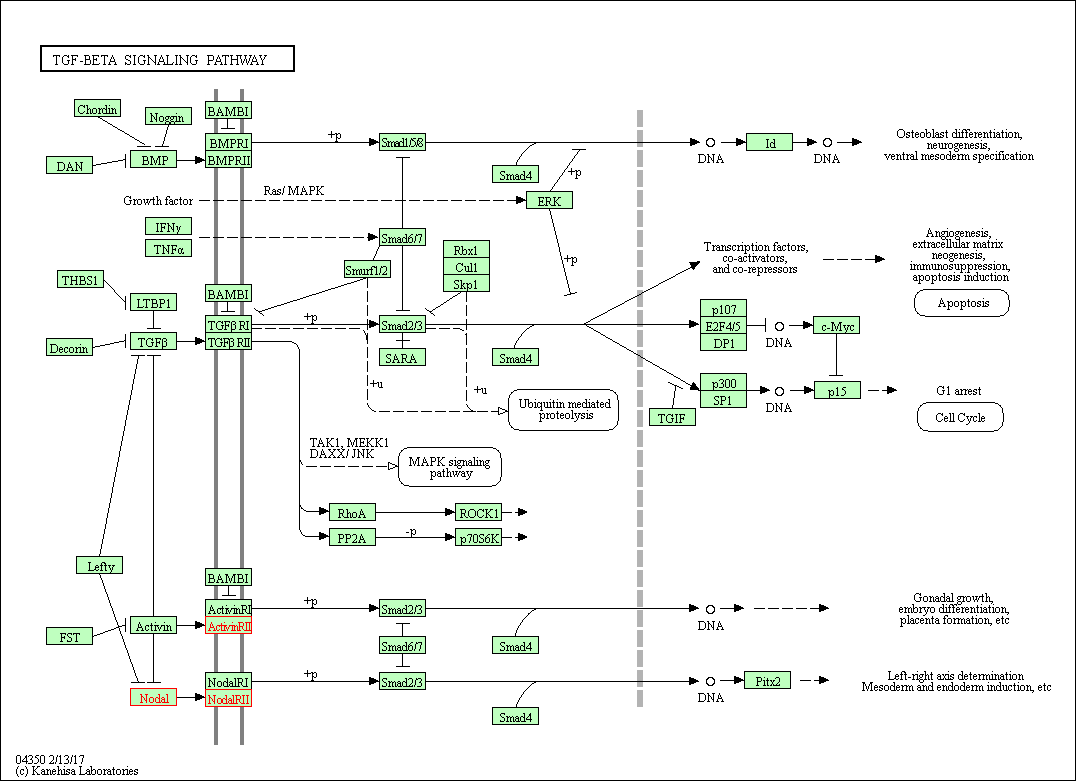

Supplement: FIGURE S5 — Output of KEGG analysis for the TGF-beta signaling pathway. Genes involved in handedness ontogenesis are highlighted in red. [file Image_5.PNG]

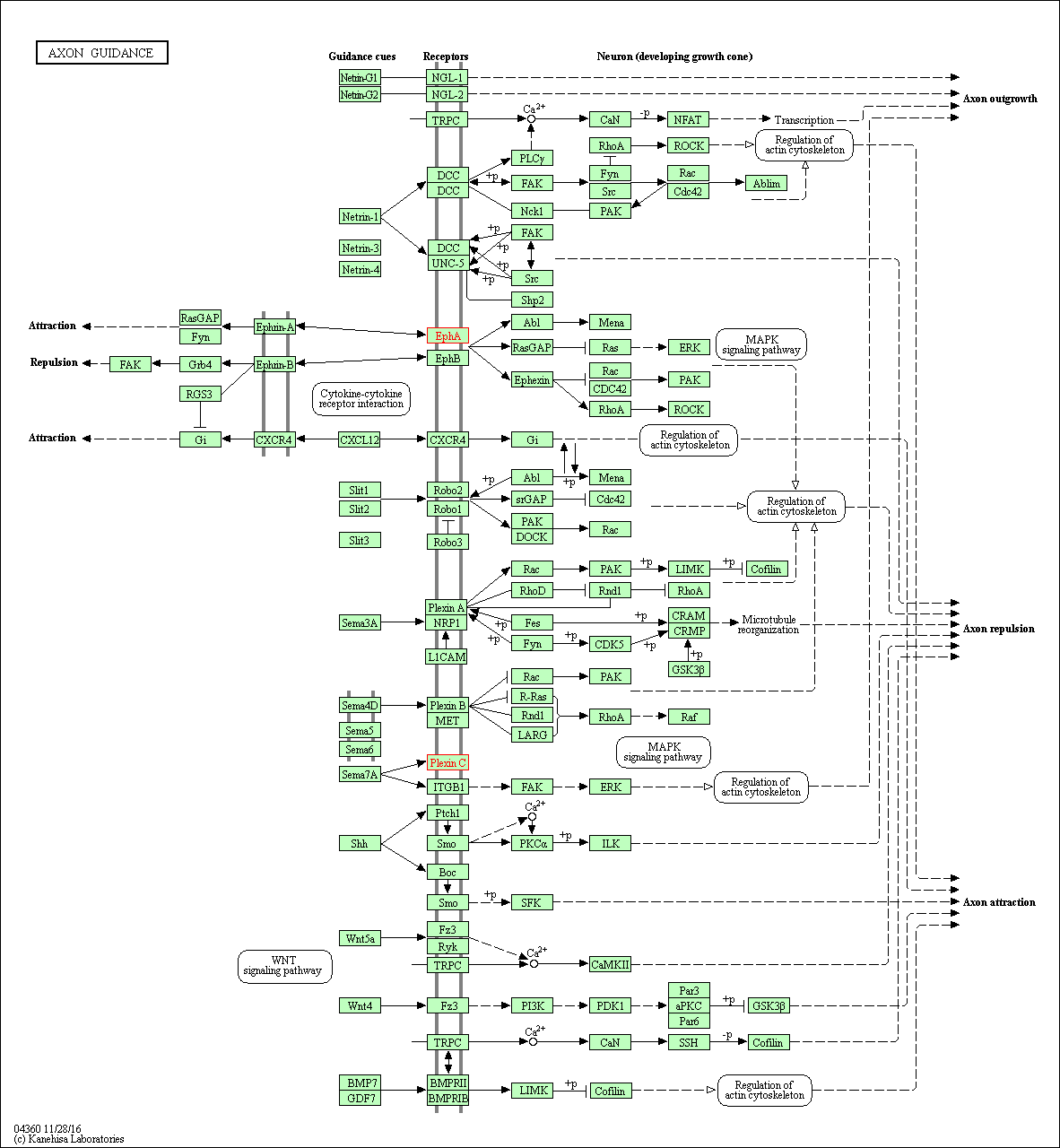

Supplement: FIGURE S6 — Output of KEGG analysis for the axon guiding pathway. Genes involved in language lateralization are highlighted in red. [file Image_6.PNG]
